# Molecular Mechanisms of Hemostasis, Thrombosis and Thrombo-Inflammation

**DOI:** 10.3390/ijms23105825

**Published:** 2022-05-23

**Authors:** Marijke J. E. Kuijpers, Johan W. M. Heemskerk, Kerstin Jurk

**Affiliations:** 1 Department of Biochemistry, Cardiovascular Research Institute Maastricht (CARIM), Maastricht University, Universiteitssingel 50, 6229 ER Maastricht, The Netherlands; jwmheem722@outlook.com; 2 Thrombosis Expertise Centre, Heart and Vascular Centre, Maastricht University Medical Centre, 6229 HX Maastricht, The Netherlands; 3 Synapse Research Institute, Kon. Emmaplein 7, 6214 AC Maastricht, The Netherlands; 4 Center for Thrombosis and Hemostasis (CTH), University Medical Center of the Johannes Gutenberg University of Mainz, Langenbeckstrasse 1, 55131 Mainz, Germany; kerstin.jurk@uni-mainz.de

In the present decade, we are seeing a rapid increase in available genetics and multiomics information on blood and vascular components of the human and mammalian circulation, involved in haemostasis, athero- and venous thrombosis, and thrombo-inflammation [1,2,3,4]. With this Special Issue, we aimed to collect state-of-the-art scientific contributions that provide novel insights into the multi-molecular interactions in hemostasis, thrombosis, and thrombo-inflammation. Particular aspects tackled are: (i) platelet and coagulation activation processes during thrombus formation (one review, five original articles), as well as (ii) interactions between platelets, neutrophils, and formation of neutrophil extracellular traps (NETs), monocytes and macrophages under pathological conditions resulting in thrombo-inflammation (two reviews, four original articles).

With regard to deepened knowledge of platelet activation mechanisms, we received a timely and extensive review by Veuthey et al. [5] which summarizes the phenotypical features of procoagulant COAT platelets. It provides an update of the molecular mechanisms leading to procoagulant COAT platelet formation. Furthermore, the authors discuss the possible drivers of the dichotomous diversification toward procoagulant versus aggregating platelets, with special attention to the platelet intrinsic factors and the external environment during thrombus formation.

During thrombus formation, several molecular pathways of platelet and coagulation activation are considered to operate simultaneously in hemostasis and thrombosis, but the spatiotemporal manner of these pathways has not been elucidated so far. Navarro et al. [6] adapted a microfluidics whole-blood perfusion assay to allow acute blockade of molecular pathways by pharmacological intervention at desired time-points during blood flow and thrombus formation. The paper shows that platelet activation processes via collagen and glycoprotein VI (GPVI-induced Syk signaling) and coagulation activation via tissue factor (TF)/thrombin (involving factor FVII and the PAR1/4 receptors) were crucial for the formation of platelet-fibrin thrombi during the first two minutes of thrombus formation. At later time-points, however, only platelet activation via PAR1/4 and integrin αIIbβ3 contributed to stabilized thrombus build-up. The stability of such platelet-rich arterial and venous thrombi depends on the presence of a core and shell region, which can be distinguished by the absence of fibrin in the shell [7], making this region more prone to breakdown. Perella et al. [8] investigated the contribution of the tyrosine kinase Syk and other signaling mediators to the stability of platelet thrombi formed on collagen or atherosclerotic plaque homogenate under shear in the absence of coagulation, resembling shell region conditions. After 7 min, post-perfusion of a Syk inhibitor enhanced the breakdown of thrombi and platelet detachment, both at room temperature and at 37 °C. This was also true for inhibitors of Src, the P2Y_12_ ADP receptor and thromboxane A_2_ (TxA_2_) formation. Furthermore, blocking of GPVI or Btk only resulted in minor thrombus breakdown. Hence, it is stated that aggregate stability on collagen is supported by Syk and Src kinases together with the secondary mediators ADP and TxA_2_. 

Another important player in thrombus formation is von Willebrand factor (vWF), which is converted to the open conformation by surface immobilization in combination with shear stress, then allowing platelet adhesion to the vessel wall via the GPIbα receptor. The study of Hrdinova et al. [9] provides further understanding of the multiple contributions of the vWF A1 domain to the thrombotic process. Stable cyclic peptides were designed in silico and chemically synthesized to specifically interfere with the opened conformation of the vWF A1 domain and the platelet GPIbα receptor. Three peptides inhibited vWF-dependent platelet adhesion and thrombus formation on collagen in whole blood under flow, although they were not as effective as a blocking anti-VWF A1 domain antibody [9]. This shows that the design of peptides based on structure results in physiologically active peptide-based inhibitors, even for intricate complexes such as GPIα-vWF A1. The results may provide a guide for the development of novel therapeutics, for example for the treatment of immune-mediated thrombocytopenic purpura. 

Also, more insight is gained into the process of GPVI shedding upon platelet activation, which is mainly mediated by ADAM10 (a disintegrin and metalloproteinase 10). ADAM10 interacts with tetraspanin membrane proteins, of which Tspan14, Tspan15, and Tspan33 are expressed in platelets. Koo et al. [10] investigated which of these tetraspanins regulate the GPVI cleavage by ADAM10, by generating CRISPR/Cas9 knockout human cell lines. They showed that Tspan15 and Tspan33 have redundant roles in GPVI cleavage, in contrast to Tspan14. Tspan15 appeared to be the dominant ADAM10 regulator for cleavage, with the extracellular region of GPVI being mechanistically crucial; a specific amino acid site close to the membrane was cleaved by the Tspan15/ADAM10 complex. 

Tspan15 can also interact with the Rho GTPase Rac1 [11], which modulates the GPVI surface expression. Neagoe et al. [12] investigated the role of Rac1 in human platelet activation and downstream signaling using the inhibitor EHT1864. This inhibitor did not affect the collagen-induced clustering of GPVI, but decreased the spreading and aggregation when platelets were stimulated by GPVI agonists. In contrast to the situation in Rac1-deficient mouse platelets, EHT1864 enhanced GPVI shedding in both resting and activated human platelets, and reduced phospholipase Cγ2 phosphorylation upon GPVI stimulation. These data suggest that the Rac1 signaling pathway operates differently between human and mouse platelets.

The other half of the papers in this Special Issue involve studies that concern aspects of (thrombo-)inflammation. We could include two state-of-the-art reviews on this topic. Mandel et al. [13] provided a clear and complete overview of the role of platelet activation in hemostasis and inflammation, including the ways how platelets interact with neutrophils, monocytes, and macrophages. Furthermore, they have zoomed in on several of these interactions, such as NET formation, and relevant pathological circumstances, including atherosclerosis, bacterial and viral infections (COVID-19), and cancer. Finally, they address the issue of how platelet-macrophage interactions contribute to platelet aging and clearance. The review of Carminita et al. [14] updates on arterial and venous thrombosis models that have been developed in different animal species using various blood vessels. In some of the mouse models, the involvement of neutrophils and/or NETs in thrombus formation and clotting has been shown. Specifically, these models involve endothelial denudation or damage by ferric chloride, and photochemical as well as ablative laser injury. In the latter model, neutrophils have been shown to be the first cells to adhere at the location of the injury, even before platelets [15]. Ligation of the vena cava as a model for deep vein thrombosis (DVT) leads to a non-ablative dysfunction of the intact endothelium. Thrombus formation in this model appears with the major cell population being more than 70% neutrophils [16]. Together, these different mouse models illustrate that neutrophils can be involved in thrombus formation via a triple coagulant mechanism. Upon NET formation, the release of DNA from neutrophils can support platelet aggregation and fibrin formation. Furthermore, the highly adhesive NET surface binds proteins from the plasma such as fibronectin and fibrinogen. Finally, the review of Carminita et al. calls upon the need for specific markers for NETs, rather than for activated neutrophils to prevent premature and unreliable conclusions.

One way to promote NET formation is by cholesterol depletion from the membrane with methyl-β-cyclodextrin (MβCD). In the tissues within the body, oxygen levels are much lower than the atmospheric O_2_ levels (normoxic), and infectious sites may show even strongly reduced oxygen levels (highly hypoxic). The study of Henneck et al. [17] showed that in mouse MβCD-induced NET formation was similar for wild-type and HIF-1α knockout mice both under hypoxic (1% O_2_) and normoxic (21% O_2_) conditions. Yet, they showed that phorbol ester-induced NET formation was reduced under hypoxic conditions. This was confirmed by experiments with human neutrophils, which were stimulated with phorbol ester, MβCD, or statins, the latter of which also depleted the cells from cholesterol.

Next to cholesterol depletion, platelet factor 4 (PF4) and high-mobility group box 1 (HMGB1), as well as histones, are strong inducers of NET formation, and the role of NETs in immunothrombosis as a major complication in sepsis and COVID-19 has been well described [13]. PF4 and HMGB1 are secreted and displayed on the surface by activated platelets. Ebeyer-Masotta et al. [18] showed that heparin-bound adsorbents Sepharose and Seraph-100 efficiently depleted activated platelets, extracellular vesicles, PF4, HMGB1, and histones/nucleosomes. This study thus suggests that heparin-functionalized adsorbents may be capable of eliminating NETs and that use of these adsorbents may be beneficial to reduce excessive NET formation in critically ill patients requiring extracorporeal blood purification.

Finally, more knowledge was gained about the role of monocytes and macrophages in thrombo-inflammatory mechanisms. A proinflammatory effect is exerted by the proprotein convertase subtilisin kexin 9 (PCSK9), independent of its action on the LDL receptors regulating plasma cholesterol. Enhancement of the local cytokine production through activation of nuclear factor kappa B (NFκB) and Toll-like receptor 4 (TLR4) may mediate this proinflammatory effect. Scalise et al. [19] discovered that PCSK9 increased the procoagulant activity and TF expression in human peripheral blood mononuclear cells (i.e., monocytes and lymphocytes) and cultured monocyte-like THP-1 cells. This effect was mediated through the TLR4 and NFκB signaling. The expression of TF, together with other components of the extrinsic coagulation pathway, also contributes to the amplification of inflammatory reactions, thus highlighting the interplay between platelets, coagulation, and inflammation [13,20]. These interactions may also affect proliferation processes in the vessel wall. Chan et al. [21] showed in a mouse model of arteriovenous fistula stenosis that both macrophage infiltration and cellular proliferation in the adventitia occur rapidly in the early stages of vascular remodeling. These results may be important for intervention strategies to prevent arteriovenous fistula maturation failure.

In conclusion, this Special Issue covers a wide range of novel molecular insights into the complex interactions in the blood and the vessel wall in physiological and pathophysiological settings. We hope that these new results may inspire investigators to develop novel research questions and will motivate to perform more research, which will lead to more new insights into the topic of molecular mechanisms of hemostasis, thrombosis, and thrombo-inflammation.
